# Biomonitoring of Oxidative-Stress-Related Genotoxic Damage in Patients with End-Stage Renal Disease

**DOI:** 10.3390/toxics12010069

**Published:** 2024-01-14

**Authors:** Yücel Yüzbaşıoğlu, Merve Hazar, Sevtap Aydın Dilsiz, Ciğdem Yücel, Mesudiye Bulut, Serdar Cetinkaya, Onur Erdem, Nursen Basaran

**Affiliations:** 1 Department of Emergency Medicine, Ankara Gülhane Training and Research Hospital, Health Sciences University, Ankara 06018, Türkiye; 2 Department of Pharmaceutical Toxicology, Faculty of Pharmacy, Ağrı İbrahim Cecen University, Ağrı 04100, Türkiye; mrvhzr56@gmail.com; 3 Department of Pharmaceutical Toxicology, Faculty of Pharmacy, Hacettepe University, Ankara 06100, Türkiye; sevtapay@hacettepe.edu.tr; 4 Department of Clinical Biochemistry, Ankara Gülhane Training and Research Hospital, Health Sciences University, Ankara 06018, Türkiye; yucelcigdem80@gmail.com; 5 Department of Nephrology, Ankara Gülhane Training and Research Hospital, Health Sciences University, Ankara 06018, Türkiye; mdmesudiyebulut@gmail.com; 6 Department of Pharmaceutical Toxicology, Gülhane Faculty of Pharmacy, Health Sciences University, Ankara 06018, Türkiye; serdarctnkaya@gmail.com (S.C.); onur.erdem@sbu.edu.tr (O.E.); 7 Department of Pharmaceutical Toxicology, Faculty of Pharmacy, Başkent University, Ankara 06790, Türkiye; anbasaran@baskent.edu.tr

**Keywords:** end-stage renal disease, dialysis, heavy metals, DNA damage, oxidative stress

## Abstract

Chronic kidney disease (CKD), a common progressive renal failure characterized by the permanent loss of functional nephrons can rapidly progress to end-stage renal disease, which is known to be an irreversible renal failure. In the therapy of ESRD, there are controversial suggestions about the use of regular dialysis, since it is claimed to increase oxidative stress, which may increase mortality in patients. In ESRD, oxidative-stress-related DNA damage is expected to occur, along with increased inflammation. Many factors, including heavy metals, have been suggested to exacerbate the damage in kidneys; therefore, it is important to reveal the relationship between these factors in ESRD patients. There are very few studies showing the role of oxidative-stress-related genotoxic events in the progression of ESRD patients. Within the scope of this study, genotoxic damage was evaluated using the comet assay and 8-OHdG measurement in patients with ESRD who were undergoing hemodialysis. The biochemical changes, the levels of heavy metals (aluminum, arsenic, cadmium, lead, and mercury) in the blood, and the oxidative biomarkers, including superoxide dismutase (SOD), catalase (CAT), glutathione peroxidase (GPx), and malondialdehyde (MDA) levels were evaluated, and their relationship with genotoxic damages was revealed. Genotoxicity, oxidative stress, and heavy-metal levels, except mercury, increased significantly in all renal patients. DNA damage, 8OHdG, and MDA significantly increased, and GSH significantly decreased in patients undergoing dialysis, compared with those not having dialysis. The duration and the severity of disease was positively correlated with increased aluminum levels and moderate positively correlated with increased DNA damage and cadmium levels. In conclusion, this study revealed that the oxidative-stress-related DNA damage, and also the levels of Al and Cd, increased in ESRD patients. It is assumed that these changes may play an important role in the progression of renal damage. Approaches for reducing oxidative-stress-related DNA damage and heavy-metal load in ESRD patients are recommended.

## 1. Introduction

Chronic Kidney Disease (CKD) is a progressive renal failure that affects more than 10% of the general population worldwide, amounting to over 800 million individuals, and has emerged as one of the most prominent causes of death and suffering in the 21st century. CKD is a general term for heterogeneous diseases that affect the structure and function of the kidney. Definition, stratification, and evaluation of CKD has been based on the Kidney Disease Improving Global Outcomes (KDIGO) guidelines since 2012. The diagnosis of CKD is made by laboratory testing, most often by estimating glomerular filtration rate (GFR) from a filtration marker, such as serum creatinine and level of albuminuria. The definition of CKD in the KDIGO report is given as the presence of kidney damage (albuminuria) or decreased kidney function (GFR < 60 mL/min/1.73 m^2^) for 3 months or longer, without clinical diagnosis. CKD is divided into five stages, according to GFR. End-stage (stage 5) renal disease (ESRD) is defined as GFR levels below 15 mL/min/1.73 m^2^, in which patients need dialysis due to the severity of nephron injury and functional loss [1,2]. As CKD continues to progress, the GFR declines, and the remaining nephrons are unable to eliminate metabolic wastes and environmental toxicants effectively from the body. As a result, the luminal and basolateral surfaces of renal tubular epithelial cells are potentially exposed to higher levels of xenobiotics, metabolic wastes, and nephrotoxic substances. DNA damage, secondary to inflammation and chronic disease, is expected to develop in ESDR [3,4].

Oxidative stress is defined as an imbalance between an excessive generation of oxidant compounds and insufficient antioxidant defense mechanisms, which leads to the tis-sue damage. It occurs naturally in humans as an important part of host defense mechanisms, but is overactivated in many pathological conditions. The most important components that are effective at the cellular level and constitute the body’s antioxidant defense are superoxide dismutase (SOD), glutathione peroxidase (GPx), catalase (CAT) enzymes and glutathione (GSH) from non-enzymatic pathways. Factors contributing to increased oxidant activity include advanced age and characteristics of the patients suffering from kidney disease, such as diabetes and uremia, for example. Hemodialysis can cause repeated increases in oxidative stress, primarily through membrane bioincompatibility and endotoxin challenge. Changes in pro-oxidant and antioxidant capacity begin in the early stages of renal damage, and are very evident in dialysis patients. However, there is some controversy whether the initiation of regular dialysis in general increases oxidative stress [5,6,7]. Free radicals and pro-oxidants produced during acute or chronic kidney injury may further aggravate the course of the disease and play a role in the pathogenesis of subsequent complications. Antioxidant enzymes, such as SOD, scavenge the superoxide anion by converting this free radical to oxygen (O_2_) and hydrogen peroxide (H_2_O_2_), thus preventing peroxynitrite production and further damage; CAT catalyzes the reaction by which H_2_O_2_ is decomposed to water and oxygen, and GPx catalyzes the reduction of various hydroperoxides (e.g., H_2_O_2_) to H_2_O, via oxidation of reduced GSH into its disulfide form (GSSH) [8].

Exposure to heavy metals may negatively alter the function of remaining functional nephrons. Environmental heavy metals such as arsenic (As), cadmium (Cd), lead (Pb), and mercury (Hg) increase nephrotoxicity at high exposure levels. Experimental evidence suggests that exposure to heavy metals may cause oxidative stress, inflammation, and lipid peroxidation in organs. Low chronic exposure to Cd, Pb, and Hg can cause both renal proximal tubular damage and a decrease in GFR [9,10,11,12]. Heavy metals including As, Cd, Pb, and Hg deplete major antioxidants of cells, particularly thiol-containing antioxidants and enzymes, which may cause an increase in production of reactive oxygen species (ROS). Cells under oxidative stress display various dysfunctions, due to lesions caused by ROS to lipids, proteins and DNA. Consequently, it is suggested that metal-induced oxidative stress may be partially responsible for the toxic effects of heavy metals in renal disease [13,14]. 

During treatment, hemodialysis patients are typically exposed to very large amounts of water two or three times per week, that is, more than 90 to 192 L of water per session. Therefore, the quality of the water used in the preparation of dialysis fluid is very important to prevent the spread of contaminants into the patient’s bloodstream [15]. Because large amounts of water are used in dialysis, even low concentrations of contaminants can pose health risks. While there were insignificant correlations between Cu, Zn and Al in dialysis fluid and blood samples, Pb concentration showed a significant correlation only in blood samples taken from men [16]. A study revealed that there was a relationship between blood lead levels and lead concentration in bones [17].

The increase in serum/blood lead levels seen in hemodialysis patients may be due, in part, to the almost complete loss of kidney function and the difficulty in removing lead during hemodialysis. Therefore, environmental lead exposure, even at low levels, can increase blood lead levels in hemodialysis patients. The blood lead levels are elevated in patients receiving chronic kidney dialysis [18].

Many studies report Cd-induced acute and chronic kidney disease, even at low exposure levels. The main molecular mechanisms of Cd toxicity are oxidative stress and mitochondrial dysfunction. Cd can accumulate particularly in individuals with kidney problems. While dialysis can help in removing some substances from the blood, its efficiency in eliminating Cd is limited. The accumulation of Cd in the kidneys impairs kidney function, which leads to increased levels of Cd in the body. The accumulation of Cd is a concern for individuals undergoing dialysis, because it is associated with various health risks, including damage to the kidneys, bones, and other organs [19,20]. 

The comet assay, a well-known technique, with its advantages of applicability, rapidity, sensitivity and economic efficiency, is frequently used to measure DNA damage, and is one of the most reliable biomarkers for indicating early biological effects in human biomonitoring studies; it is therefore accepted by various governmental regulatory agencies [21]. 8-Hydroxy-2′-deoxyguanosine (8-OHdG), one of more than 20 oxidative-base-damage products caused by reactive oxygen species, is formed as a result of the attack of the hydroxyl radical on the eighth carbon atom of the guanine base in DNA. This base is accepted as a direct measure of oxidative DNA damage [22,23]. 

Within the scope of this study, the changes in genotoxicity and oxidative stress parameters in ESRD patients, both those undergoing and those not undergoing hemodialysis, were evaluated and compared with healthy controls, in detail. DNA damage in the peripheral lymphocytes of the subjects was determined by the alkaline comet assay. Oxidative stress parameters including SOD, CAT, GPx, malondialdehyde (MDA) levels, and 8-OHdG (indicating the oxidative-stress-related DNA damage) were measured in the plasma samples, using ELISA kits. The levels of heavy metals including aluminum (Al), As, Cd, Pb, and Hg were also determined, using inductively coupled plasma mass spectrometry (ICP-MS). To our knowledge, our study is the first to evaluate genotoxicity, oxidative stress, heavy-metal levels, and biochemical changes in detail, in ESRD patients with and without dialysis. 

## 2. Materials and Methods

### 2.1. Patients and Study Design

The patient group with ESRD consisted of 43 patients undergoing hemodialysis and 31 patients not undergoing hemolysis. The control group consisted of 40 healthy volunteers, similar to the patient group in terms of age, gender, lifestyle, smoking habits and alcohol use, and without a history of chemical exposure (Table 1).

Patients diagnosed with ESRD in the research group were selected from the patients who were admitted to the Nephrology Clinic at Gülhane Training and Research Hospital, located in Ankara, Türkiye. ESRD patients undergoing dialysis were undergoing dialysis regularly, three times a week.

The criteria for inclusion and exclusion from the study are as follows:

The ESRD was diagnosed by a nephrologist, according to the KDIGO (stage 4 and stage 5) definition [2,24]. Patients over the age of 18, who were not pregnant and agreed to participate in the study, were included.

Individuals with liver dysfunction, acute or chronic infection, heart failure, acute kidney and thyroid disease, vasculitis, connective tissue disease, or malignancy, who were using immunosuppressive agents or receiving radiotherapy or chemotherapy treatment, or who had a history of drug use (statins, antioxidants or vitamins) were excluded from the study.

All volunteers were instructed with information about the aim of the study, and their written consent was obtained. A detailed questionnaire form including age, gender, smoking and alcohol use, medication and vitamin use, health status, and history of diseases related to kidney functions was given to the individuals participating in the study, before the sample collection.

This study was approved by the local ethics commission of Hacettepe University Non-Interventional Clinical Research Ethics Committee, in accordance with the 1964 Declaration of Helsinki (Date: 29 June 2021; No. GO 21/884).

### 2.2. Sample Preparation

A total of 10 mL peripheral blood sample was collected from each volunteer, as a biological sample. All blood samples were maintained at +4 °C and processed within two hours. The timing of blood sample collection for dialysis patients was within 1 h after the dialysis process ended.

For the analysis of hemogram parameters, 2 mL peripheral blood sample was taken, and collected in EDTA tubes. Hemograms and plasma biochemical parameters were measured immediately in the Medical Biochemistry laboratory of the University of Health Sciences, Faculty of Medicine. A total of 8 mL of peripheral blood sample from each volunteer was taken, and collected in a sodium heparin tube. It was delivered to Hacettepe University Faculty of Pharmacy Toxicology Department Research Laboratory within 6 h in a cold transport container, and protected from light. A total of 1 mL of heparinized blood sample was used for the analysis of DNA damage. The remaining 6 mL and 1 mL heparinized blood samples were used for the analysis of oxidative-stress parameters (SOD, CAT, GPx, GSH, MDA, 8-OHdG) and heavy-metal analyses, respectively. Plasma samples were frozen in aliquots at −20 °C and stored at −80 °C, until the day of analysis.

### 2.3. Determination of Hemogram

The levels of white blood cell (WBC), red blood cell (RBC), hemoglobin (Hb), platelet (PLT), hematocrit (HCT), mean cell volume (MCV), mean cell hemoglobin (MCH), mean cell hemoglobin concentration (MCHC), RBC-distribution-width standard deviation (RDW-SD), red cell distribution width (RDW-CV), platelet distribution width (PDW), mean platelet volume (MPV), neutrophils, monocytes, lymphocytes, eosinophils, and basophils were analyzed using the turbidimetric method, using the Sysmex XN-2000 (Sysmex Europe GMBH, Bornbarch 1, 22848, Norderstedt, Germany) hematology autoanalyzer. The samples were analyzed in duplicates. Results were given as mean ± standard deviation (range). The units were as follows: 10^3^/µL for WBC, PLT; 10^6^/µL for RBC; g/dL for Hb and MCHC; femtoliter (fL) for MCV, RDW-SD, PDW and MPV; picogram (pg) for MCH; % for HCT, RDW-CV, neutrophils, monocytes, lymphocytes, eosinophils, and basophils.

### 2.4. Determination of Biochemical Parameters

The liver function parameters (AST, ALT), glucose, uric acid, sodium, and potassium in plasma samples were analyzed using the Roche Cobas C702 Chemistry Analyzer (Roche Diagnostics, Istanbul, Türkiye). AST, ALT, glucose and uric acid were measured using the kinetic enzymatic colorimetric method. Sodium and potassium were determined using the indirect ISE (ion selective electrode) method. The samples were studied in duplicates. Results were given as mean ± standard deviation (range). The units were as follows: U/L for AST and ALT; mg/dL for glucose and uric acid; mEq/L for sodium and potassium.

### 2.5. Determination of Renal Function

The creatinine was determined using the kinetic colorimetric Jaffe method, using a Roche Cobas C702 Chemistry Analyzer (Roche Diagnostics, Basel, Switzerland) [25]. In alkaline solution, creatinine creates a yellow–orange color with picrate. The rate of dye formation is directly proportional to the creatinine concentration in the sample. The linear detection range of the method was 0.17–24.9 mg/dL (15–2200 µmol/L) in serum and 4.2–622 mg/dL (375–55,000 µmol/L) in urine. The examples were studied in duplicate. Results are given as mean ± standard deviation (range). The unit of creatinine was mg/dL.

The urea levels in the samples were determined using the kinetic method, involving urease and glutamate dehydrogenase and using the Roche Cobas C702 Chemistry Analyzer (Roche Diagnostics, Basel, Switzerland). In the first step, urea is converted into ammonium and carbonate by the urease enzyme. In the second reaction, 2-oxoglutarate combines with ammonium and is converted to glutamate by the enzyme glutamate dehydrogenase. The decrease in NADH, the reaction cofactor, is directly proportional to the amount of urea, and is measured photometrically at a wavelength of 340 nm. The linear detection range of the method in serum was 3.0–240 mg/dL (0.5–40 mmol/L).

The GFR, an indicator of renal function, was calculated using the CKD-EPI equation—including age, sex, ethnicity, and serum creatinine [26,27]. The CKD-EPI equation is GFR(mL/min/1.73 m^2^) = 141 (if female) or 144 (if male) × min(Scr/κ, 1)^α^ × max(Scr/κ, 1)^−1.209^ × 0.993^Age(years)^ × 1.018 (if female)_1.159 (if black), where Scr is serum creatinine in mg/dL, κ is 0.7 for females and 0.9 for males, α is −0.329 for females and −0.411 for males, min indicates the minimum of Scr/κ or 1, and max indicates the maximum of Scr/κ or 1.

### 2.6. Determination of Heavy-Metal Levels

The heavy-metal levels, including Al, As, Cd, Hg and Pb in plasma samples were determined using the inductively coupled plasma mass spectrometry (ICP-MS) technique, with Thermo Scientific ICAP Qc Series (Bremen, Germany). The determination of heavy metals was carried out in the laboratory of the Gülhane Faculty of Pharmacy, University of Health Sciences.

Nitric acid SUPRAPURE^®^ grade (65%) (Merck KGaA, Darmstadt, Germany) was used in the dilution of the samples. All aqueous solutions were prepared with deionized water, obtained using an ultrapure water system (Aqua Nova Hepta Distilled, resistivity 0.34 MΩ-cm, Kristianstad, Sweden). All materials used in the analytical processes were cleaned by soaking in HNO3 (10% *v*/*v*) for one day, rinsing with ultrapure water four times, and drying in an oven at 40 °C. Samples (1 mL plasma samples) were stored in capped polypropylene tubes at +4 °C until analysis. Stock solutions of the analytes (1 g/L each) were obtained from Merck (Merck KGaA, Darmstadt, Germany). All prepared standard solutions were stored in high-density polypropylene bottles. Standard solutions were prepared freshly by diluting stock solutions in 2% HNO3. All blanks, calibration standards, quality control samples and samples were spiked with gold (Au), to a concentration of 200 µg/L.

Each sample was applied directly to the device after diluting 1:20 with 2% Nitric acid prepared with 65% Suprapure Nitric acid. Device settings are as follows: Peristaltic pump speed, 40 rpm; Tubing to pump, orange/green tubing for sample transport; Nebulizer, PFA-ST; RF power, 1550 W; Cooling gas flow, 14 L/min; Auxiliary gas flow, 0.8 L/min; Nebulizer gas flow, 0.97 L/min; Dwell time, 0.001–0.02 microseconds.

The Qtegra™ Intelligent Scientific Data Solution™ version 1 (ISDS) (Thermo Fisher Scientific, Bremen, Germany) software program was used in the analysis of ICP-MS data. The measurements were repeated three times.

### 2.7. Determination of Oxidative-Stress-Related Parameters

The levels of SOD, CAT, GPx, GSH, MDA, 8-OHdG, which are oxidative-stress indicators, were determined spectrophotometrically, using ELISA test kits from ELK Biotechnology Co., Ltd. (Denver, CO, USA) and Shanghai SunRed Biological Technology Co., Ltd. (Baoshan District, Shanghai, China) with manufacturer’s directions, at 450 nm. Protein concentrations of the plasma samples were determined spectrophotometrically, using Pierce™ BCA Protein Assay Kit (Thermo Fisher Scientific, Waltham, MA, USA) with manufacturer’s directions, at 562 nm. Additional reagents were purchased from Sigma-Aldrich (St Louis, MO, USA). For spectrophotometric measurements, a SpectraMax M2 (Molecular Devices, Sunnyvale, CA, USA) was used, and for quantification, SoftMax Pro Software 7.1 (Molecular Devices) were used. The samples were studied in duplicate. The units are as follows: ng/mL for SOD, CAT, GPx, 8-OHdG; nmol/mL for GSH, MDA.

### 2.8. Determination of DNA Damage

The alkaline comet assay (the single-cell gel electrophoresis technique) was performed to determine DNA damage [28,29,30]. The peripheral blood mononuclear cells from heparinized blood samples were isolated using Histopaque^®^-1077 (Sigma-Aldrich (St Louis, MO, USA) and washed with PBS. Cell viability checked with the trypan blue exclusion test was higher than 95% in all cases. After the isolation of the lymphocytes, the cell concentrations were adjusted to approximately 2 × 10^5^ cells/mL in this buffer. The cells (10^4^ cells/slides) in 1% low-melting-point (LMP) agarose were embedded on the slides pre-coated with 1% normal-melting-point (NMP) agarose, and lysed with fresh, ice-cold lysis solution (2.5 M NaCl, 100 mM EDTA, 100 mM Tris, 1% sodium sarcosinate, pH 10.00), with 1% Triton X-100 and 10% DMSO for 1 h at 4 °C, to form nucleoids containing supercoiled loops of DNA linked to the nuclear matrix. After the lysation step, the slides were drained and kept in the alkaline electrophoresis solution (1 mM sodium EDTA and 300 mM NaOH, pH 13.00) for 20 min at 4 °C, to allow unwinding of the DNA and expression of alkali-labile damage. Electrophoresis, with a current of 25 V (300 mA), was then applied for 20 min at 4 °C. After the electrophoresis, the slides were subsequently washed with distilled water to remove residues of detergents and salts, and then neutralized for 15 min in Tris buffer, pH 7.50. The slides were left in 50%, 75% and 98% alcohol for 5 min, consecutively. The dried microscopic slides were stained with ethidium bromide (20 μg/mL in distilled water) and covered with a cover-glass. In order to determine the degree of DNA damage after electrophoretic migration of DNA fragments in the agarose gel, the image analysis of the slides was performed using a green-light fluorescence microscope (LeicaVR M205 FCA, Wetzlar, Germany) connected to a charge-coupled device camera and personal computer-based analysis system Comet Assay IV™ version 1 software (In-Stem-Perceptive Instruments Ltd., Suffolk, Halstead, UK). One hundred cells from each of duplicate slides were examined at 400× magnification by a well-trained comet specialist. The extent of DNA damage was expressed as the percentage of DNA in the tail (tail intensity).

### 2.9. Statistical Analysis

Statistical evaluation of the data set was carried out using IBM^®^ SPSS Statistics version 23.0 software for Windows. The samples were studied in triplicate. The results were presented as mean ± standard deviation (SD) (min–max) and the percentage number of cases (%) for continuous variables and categorical variables, respectively.

Statistical differences between groups with normal distribution were determined using the one-way analysis of variance (ANOVA) test. Post hoc analysis of group differences was performed with the least-significant-difference (LSD) test. Statistical differences between groups without normal distribution were analyzed using he Mann–Whitney U test for two groups and the Kruskal–Wallis test for more than two groups. The z-test was applied for statistical analysis of categorical values.

The homogeneity of variance was tested using the Levene test. The Kolmogorov–Smirnov test was used to determine the normality of distribution. The magnitude of linear relationship was calculated using Pearson correlation analysis. A *p*-value of less than 0.05 was considered as statistically significant.

## 3. Results

### 3.1. Characteristics of the Study Groups

The characteristics of the patients and healthy controls, including age, gender, body mass index (BMI), smoking and alcohol habits, duration of renal disease, and the findings regarding to health status are given in Table 1.

The mean ages of ESRD patients undergoing dialysis, patients who were not on dialysis, and the control group were 59.43 ± 16.88 (32–83) years, 67.55 ± 17.69 (37–87) years, and 61.73 ± 18.06 (33–87) years, respectively. The mean BMI (kg/m^2^) of ESRD patients undergoing dialysis, ESRD patients not on dialysis, and the control group were 2.46 ± 0.48 (0.53–3.29), 2.79 ± 0.52 (1.85–4.30), and 2.74 ± 0.53 (1.76–4.10), respectively. The percentages of males in patients undergoing dialysis, patients without dialysis, and the control group, were 62.79%, 45.16%, and 51.22%, respectively. There were no statistically significant differences between the patients and the healthy controls in terms of age, BMI, and gender (Table 1).

It was determined that mean cigarette consumption per day of ESRD patients undergoing dialysis, ESRD patients not on dialysis, and the control group were 2.09 ± 6.00 (10–20) cigarettes/day (n = 5, 11.53%), 3.33 ± 7.58 (20–20) cigarettes/day (n = 5, 16.13%), and 2.50 ± 8.09 (20–40) cigarettes/day (n = 4, 12.43%), respectively, and the differences between the groups were not statistically significant. All individuals in the patient and control groups stated that they did not consume alcohol at all (Table 1).

The duration of renal disease in ESRD patients undergoing dialysis and ESRD patients without dialysis were 8.16 ± 3.69 (2–16) years and 5.07 ± 1.70 (2–8) years, respectively (Table 1).

Diabetes mellitus was present as an additional disease in 53.4% of ESRD patients undergoing dialysis, with hypertension in 74.5% and heart failure in 93.03%. Diabetes mellitus was present as an additional disease in 48.4% of ESRD patients without dialysis, with hypertension in 66.5% and heart failure in 90.33% (Table 1).

### 3.2. Hemogram and Biochemical Parameters

The hemograms and biochemical parameters of the study groups are presented in Table 2 and Table 3, respectively.

There was no difference between the study groups in terms of the hemogram values, including WBC, PLT, MCV, MCH, MCHC, RDW-SD, RDW-CV, MPV, NEU, MO, EOS, and BASO (*p* > 0.05) (Table 2).

The values of RBC (1.25 fold), Hb (1.23 fold), HCT (1.23 fold), and LYM (1.54 fold) in ESRD patients undergoing dialysis and the values of RBC (0.90 fold vs. control), Hb (1.12 fold vs. control), and HCT (1.12 fold vs. control) in ESRD patients without dialysis decreased significantly when compared with healthy controls (*p* < 0.05) (Table 2).

There was no difference in all hemogram parameters between ESRD patients undergoing dialysis and the patients without dialysis (*p* > 0.05) (Table 2).

The difference between the study groups in AST values was not significant (*p* > 0.05). ALT levels significantly decreased in ESRD patients on dialysis (1.83 fold) compared with healthy controls (*p* < 0.05); however, there was no difference in ALT levels between the patients with ESRD and the patients without dialysis or between the patients without dialysis and the healthy control group (*p* > 0.05) (Table 3).

There was no difference in glucose, natrium (Na), and potassium (K) levels between the study groups (*p* > 0.05) (Table 3).

### 3.3. Renal Function Parameters

The renal function parameters of the study groups are given in Table 4. Serum creatinine levels were significantly higher in ESRD patients undergoing dialysis (4.8 fold) and in ESRD patients not on dialysis (1.87 fold) than in the control group. However, the creatinine levels (2.34 fold) of ESRD patients undergoing dialysis were significantly higher than those of ESRD patients not undergoing dialysis (*p* < 0.05) (Table 4).

The urea levels were significantly higher in ESRD patients on dialysis (2.37 fold) and in ESRD patients not on dialysis (1.93 fold) than in the control group. However, the urea levels (1.23 fold) of ESRD patients undergoing dialysis were significantly higher than those of ESRD patients not undergoing dialysis (*p* < 0.05) (Table 4).

The GFR values were significantly lower in ESRD patients on dialysis (0.18 fold) and in ESRD patients not on dialysis (0.41 fold) than in the control group. However, the GFR values (0.43 fold) of ESRD patients undergoing dialysis were significantly lower than those of ESRD patients not undergoing dialysis (*p* < 0.05) (Table 4).

### 3.4. Heavy-Metal Levels

The levels of heavy metals, including Al, Cd, Pb, As, and Hg of the study groups are given in Table 5.

Al levels of healthy controls were lower than the detection limit; however, the Al levels were 3.58 ± 0.72 (2.94–5.50) ppm and 3.80 ppm in 47.8% (n = 11) of ESRD patients on dialysis, and 3.57% (n = 1) of ESRD patients without dialysis, respectively. The levels were significantly higher in patient groups compared with healthy controls (*p* < 0.05), while there was no significant difference in Al levels between the patient groups (*p* > 0.05) (Table 5). There was no strong correlation between GFR and Al levels. When all patients with and without dialysis were included, a moderately negative correlation was found between GFR values and Al levels (r = −0.331). When correlation analysis was performed individually between patient groups and GFR, a moderately significant negative correlation was found only between Al levels and GFR of patients not undergoing dialysis (r = −0.495). We can say that the negative correlation between Al levels and GFR in the patient group originates from the patient group that is not on dialysis.

The Cd levels were 8.07 ± 15.76 (0.05–49.36) ppm in 73.9% (n = 17) of the patients on dialysis, 0.67 ± 0.98 (0.05–4.25) ppm in 75.0% (n = 21) of the patients without dialysis, and 0.16 ± 0.12 (0.05–0.45) ppm in 53.1% (n = 17) of the healthy controls. Cd levels in ESRD patients on dialysis were significantly higher than in ESRD patients without dialysis (12.14 fold) and the healthy control group (50.43 fold) (*p* < 0.05). There was no significant difference in Cd levels between ESRD patients without dialysis and the healthy controls (*p* > 0.05). There was no strong correlation between GFR and Cd levels. A very low negative correlation was found between Cd levels and GFR of patients undergoing dialysis (r = −0.06) and a low negative correlation was found between Cd levels and GFR of patients not undergoing dialysis (r = −0.16). When all patients with and without dialysis were included, a low negative correlation was found between GFR values and Cd levels (r = −0.19).

The Pb levels were 13.57 ± 9.03 (3.34–35.29) ppm in 43.5% (n = 10) of the patients on dialysis, and 20.41 ± 15.11 (5.67–52.25) ppm in 39.3% (n = 11) of the patients without dialysis. It was detected as 0.38 ppm in 3.12% (n = 1) of the healthy control group. Pb levels were significantly higher in ESRD patients on dialysis (35.71 fold) and in ESRD patients without dialysis (53.71 folds) than in the healthy controls (*p* < 0.05). There was no significant difference between ESRD patients on dialysis and ESRD patients without dialysis (*p* > 0.05). There was no strong correlation between GFR and Pb levels. A very low negative correlation was found between Pb levels and GFR of patients undergoing dialysis (r = −0.02), and a low positive correlation was found between Pb levels and GFR of patients not undergoing dialysis (r = 0.22). When all patients with and without dialysis were included, a low negative correlation was found between GFR values and Pb levels (r = −0.19).

The As levels were 4.35 ± 0.93 (2.85–6.35) ppm in 100% of the patients (n = 23) on dialysis, 3.86 ± 1.67 (2.19–10.45) ppm in 92.9% (n = 26) of the patients without dialysis, and 2.35 ± 0.78 (1.23–5.46) ppm in 100% of the healthy control group (n = 32). As levels were significantly higher in ESRD patients on dialysis (1.85 fold) and in ESRD patients without dialysis (1.64 fold) than in the healthy control group (*p* < 0.05). There was no significant difference between ESRD patients on dialysis and ESRD patients without dialysis (*p* > 0.05). There was no strong correlation between GFR and As levels. A moderate positive correlation was found between As levels and GFR of patients undergoing dialysis (r = 0.42), and a low positive correlation was found between As levels and GFR of patients not undergoing dialysis (r = 0.10). When all patients with and without dialysis were included, a very low negative correlation was found between GFR values and As levels (r = −0.01).

Since Hg levels in the samples of the study groups were below the detection limits, no significant differences were found between the groups.

### 3.5. Oxidative-Stress-Releated Parameters

The oxidative-stress parameters, including SOD, CAT, GPx, GSH, MDA, and 8-OHdG are given in Table 6.

The SOD levels of ESRD patients on dialysis (2.90 fold) and ESRD patients without dialysis (1.65 fold) were significantly higher than in the healthy controls (*p* < 0.05). SOD levels of ESRD patients on dialysis (1.75 fold) were significantly higher than in ESRD patients without dialysis (*p* < 0.05) (Table 6).

The CAT levels of ESRD patients on dialysis (1.36 fold) and ESRD patients without dialysis (1.49 fold) were significantly higher than in the healthy controls (*p* < 0.05). There was no difference in CAT levels between ESRD patients on dialysis and ESRD patients without dialysis (*p* > 0.05) (Table 6).

The GPx levels of ESRD patients on dialysis (1.41 fold) and ESRD patients without dialysis (1.46 fold) were significantly higher than in the healthy controls (*p* < 0.05). There was no difference in GPx levels between ESRD patients on dialysis and ESRD patients without dialysis (*p* > 0.05) (Table 6).

The GSH levels of ESRD patients on dialysis (3.13 fold) and ESRD patients without dialysis (2.33 fold) were significantly lower than in the healthy controls (*p* < 0.05). There was no difference in GSH levels between ESRD patients on dialysis and ESRD patients without dialysis (*p* > 0.05) (Table 6).

The MDA levels of ESRD patients on dialysis (2.86 fold) and ESRD patients without dialysis (1.83 fold) were significantly higher than in the healthy controls (*p* < 0.05). MDA levels of ESRD patients on dialysis (1.56 fold) were significantly higher than in ESRD patients without dialysis (*p* < 0.05) (Table 6).

The 8-OHdG levels of ESRD patients on dialysis (4.09 fold) and ESRD patients without dialysis (2.06 fold) were significantly higher than in the healthy controls (*p* < 0.05). 8-OHdG levels of ESRD patients on dialysis (1.98 fold) were significantly higher than in ESRD patients without dialysis (*p* < 0.05) (Table 6).

### 3.6. DNA Damage from the Alkaline Single-Cell Gel Electrophoresis Technique

**Figure 1:**
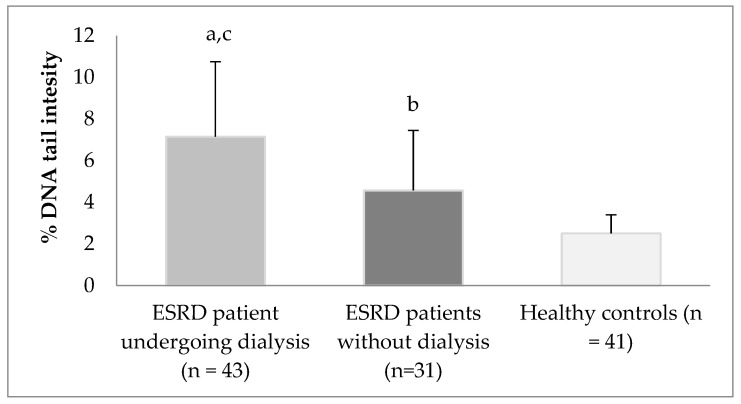
DNA damage in the lymphocytes of the study groups. DNA damage is expressed as % DNA tail intensity. The values are given as mean ± standard error mean (min–max). ^a^ *p* < 0.05, ESRD patients on dialysis compared with healthy controls; ^b^ *p* < 0.05, ESRD patients without dialysis compared with healthy controls, ^c^ *p* < 0.05, ESRD patients on dialysis compared with ESRD patients without dialysis.

DNA damage expressed as% DNA tail intensity (% of DNA in the tail) in the lymphocytes is shown in Figure 1. DNA damage increased significantly in all ESRD patients with or without dialysis; moreover, DNA damage in ESRD patients on dialysis increased more dramatically than in the control group (*p* < 0.05).

DNA tail intensity in ESRD patients on dialysis and ESRD patients without dialysis were 2.85 fold and 1.82 fold higher than in the healthy controls, respectively (*p* < 0.05) (Figure 1). DNA tail intensity in ESRD patients on dialysis was 1.56 fold higher than in ESRD patients without dialysis (*p* < 0.05) (Figure 1).

### 3.7. Relationships between Duration of Renal Disease and Dialysis with Heavy-Metal Levels, Oxidative Stress, and DNA-Damage Parameters

The ESRD patients were grouped according to their disease duration (short: 2–5 years, medium: 6–9 years, and long: 10–16 years), and the results regarding their relationships with heavy-metal levels, oxidative stress and DNA-damage parameters are given in Table 7.

The Al and Cd levels of the patients with long duration were significantly (1.20 fold and 7.66 fold, respectively) higher than in the patients with short duration (*p* < 0.05). There was no difference in Al levels between the patients with short and medium duration and between the patients with medium and long duration (*p* > 0.05). There was no difference in Pb and As levels among all groups (*p* > 0.05) (Table 7).

The MDA levels of the patients with long duration were significantly higher than the patients with short and medium duration (1.56 fold and 1.57 fold, respectively) (*p* < 0.05). There was no difference in MDA levels between the patients with short and medium duration (*p* > 0.05). 8-OHdG levels of the patients with long duration were significantly higher than in the patients with short and medium duration (1.84 fold and 1.64 fold, respectively) (*p* < 0.05). There was no difference in 8-OHdG levels between the patients with short and medium duration (*p* > 0.05). There were no differences in SOD, CAT, GPx, or GSH levels among all groups (*p* > 0.05) (Table 7).

DNA damage in the lymphocytes of the patients with long duration was significantly higher than in the patients with short and medium duration (1.48 fold and 1.52 fold, respectively) (*p* < 0.05). There was no difference in DNA damage between the patients with short and medium duration (*p* > 0.05) (Table 7).

There was a strong positive relationship between the duration of disease and Al levels (r = 0.52), a moderate positive relationship between the duration of disease and the levels of DNA damage (r = 0.31) and Cd (r = 0.45), and a weak or very weak relationship between the duration of disease and the other parameters (SOD, r = 0.04; CAT, r = 0.05; GPx, r = 0.07; GSH, r = −0.17, MDA, r = 0.12; 8-OHdG, r = 0.14; Pb, r = −0.14; As, r = 0.25).

There was a moderate positive relationship between dialysis duration and Al (r = 0.48), Cd (r = 0.39), SOD (r = 0.33), GSH (r = 038), 8-OHdG (r = 0.40), and DNA damage (r = 0.36) levels. A moderate negative relationship was determined between dialysis duration and GSH levels (r = −038). There was a weak or very weak relationship between dialysis duration and other parameters, including CAT (r = 0.14), GPx (r = 0.16), Pb (r = −0.23), and As (r = 0.19).

## 4. Discussion

Renal functions have been lost irreversibly in ESRD patients who need renal replacement therapies like hemodialysis to maintain their vital functions. An increase in oxidative stress and heavy-metal levels are two main factors which have been suggested to cause functional loss in nephrons. However, the pathophysiological mechanism underlying these detrimental effects is not fully understood. Moreover, the effects of hemodialysis on oxidative stress and heavy-metal exposure are other points to be clarified in ESRD patients [9,10,11,12]. Therefore, in this study, we focused on the evaluation of the changes in heavy-metal levels, DNA damage, and oxidative-stress markers among ESRD patient, both in those undergoing dialysis and in those not undergoing dialysis, compared with healthy controls.

Anemia is an expected clinical outcome in ESRD. In our study, the levels of RBC, Hb, and HTC in the patients were found to be statistically lower than in the healthy controls. The levels of these three parameters in ESRD patients on dialysis were lower than in ESRD patients who were not undergoing dialysis. The cutoff levels for anemia were defined as being Hb levels of 12 g/dl in women and 13 g/dl in men, using the World Health Organization criteria [31]. In the large ESRD cohort study by Saraf et al. [32], among 3919 participants, 1859 (47%) had anemia at baseline. A high percentage of approximately half of ESRD patients in the United States have been reported to suffer from anemia due to erythropoietin deficiency, resulting from impairment of erythropoietin synthesis by accumulated uremic toxins [33]. Our findings are in concordance with these studies, as we found Hb levels to be 10.44 g/dL in ESRD patients undergoing dialysis and 11.44 g/dL in ESRD patients not undergoing dialysis. These findings support the presence of a deeper anemia in ESRD patients who receive hemodialysis.

The ESRD patients are an important group, with chronic co-morbidities, and they require regular laboratory examinations for their follow-up. Hepatic diseases are common among ESRD patients [34,35]. Therefore, AST and ALT, the most common liver functions, were evaluated in our study. We found that ALT levels of ESRD patients undergoing dialysis were significantly lower than in healthy controls; however, there were no significant differences between the patients on dialysis and the patients not undergoing dialysis, or between the patients without dialysis and the healthy controls. The study carried out by Ray et al. [36] showed that AST and ALT levels were lower in ESRD patients. Other previous studies also showed that ALT levels in patients with CKD were lower than in healthy controls [35,37,38]. The fact that ALT levels decreased in ESRD patients receiving hemodialysis treatment in our study is also consistent with these results. These findings show that liver function is deeply impaired in ESRD patients undergoing dialysis, as ALT is the main enzyme which is specific to hepatic function loss. A low serum-ALT level could be due to water retention and hemodilution in ESRD patients, especially in those undergoing dialysis. Urea and creatinine values are checked before dialysis in ESRD patients, and significantly higher urea and creatinine values and lower GFR values were detected in ESRD patients undergoing dialysis compared with other groups, as expected.

It has been shown that there is a positive relationship between chronic kidney disease and heavy-metal levels (Cd, Pb, Hg) from a cross-sectional study by the National Health and Nutrition Examination Survey 2011–2020, which included 12,412 participants [39].

Al has been widely found in dialysate fluids. Neurological symptoms related to Al were reported in dialysis patients [40]. In 2003, the National Kidney Foundation Kidney Disease Quality Outcomes Initiative (NKF KDOQI) opined that serum-Al concentrations should be less than 20 µg/L (ppb) to prevent the progressive accumulation of Al in dialysis patients and patients with severe chronic kidney disease [41]. ESRD patients are at risk of Al toxicity, due to exposure from dialysis solutions, medications, and, sometimes, even from drinking water. Al can accumulate in the body because impaired kidney function hinders its excretion. In our study, the levels of Al were lower than detection limit in the healthy controls, but it was detected in 47.8% and 3.57% of the patients with dialysis and without dialysis, respectively. Al levels were 3.58 ± 1.88 ppb (2.94–5.50) in patients undergoing dialysis, while the mean value was 3.80 ± 0.73(3.80–3.80) ppb in patients not undergoing dialysis. Therefore, the Al levels were found to be significantly higher than in the healthy controls. Between two patient groups, no statistically significant difference was detected among Al levels. A moderately negative correlation between GFR values and Al levels (r = −0.331) was also observed in our patient groups.

Cd is a major environmental pollutant and nephrotoxicant. ESRD patients are a high-risk group, as following chronic exposure to Cd, approximately 50% of the metal accumulates in the kidney [42,43]. Cd-induced GFR reduction was greater in those with diabetes, hypertension, or both [44]. It was reported that blood Cd levels ≥0.53 μg/L were associated with 2.21-fold increases in the risk of a reduced GFR from the survey by U.S. NHANES 2011–2012 (n = 1545) [45]. In our study, Cd levels were similar in ESRD patients not on dialysis and in healthy controls. But in ESRD patients undergoing dialysis, Cd levels were significantly higher than in the other two study groups, and the levels were found to be as high as 7.94 ± 13.62 (0.05–49.36) ppb. The American Conference of Governmental Industrial Hygienists defines the biological exposure index as a blood Cd level that has been shown to be associated with renal function at chronic exposure of 5 ppb [46]. Therefore, in our study, Cd levels are also in concordance with these toxic levels in ESRD patients undergoing dialysis, supporting the toxic accumulation of Cd in kidneys.

Pb is a toxic metal which has serious consequences on the nervous, circulatory, skeletal, renal, hematopoietic, and endocrine systems. Exposure to Pb may also result in nephropathy and renal adenocarcinoma [47]. Exposure to low levels of Pb early in life has been shown to lead to glomerular hypertrophy. Chronic exposure to Pb may lead to progressive tubulointerstitial nephritis, which leads to CKD. One of the primary cellular effects of exposure to Pb is the induction of oxidative stress in renal cells. Exposure to Pb has also been shown to lead to lipid oxidation and DNA fragmentation [48]. The results of our study showed that Pb levels were elevated in both ESRD patient groups compared with healthy controls. These high concentrations of Pb in ESRD patients might be one of the reasons for increased oxidative stress and DNA damage in our patient groups. Chronic inflammation is a significant contributor to the promotion of oxidative stress. The presence of proinflammatory factors in CKD leads to an increase in oxidative stress, while, at the same time, the disturbed redox balance in the body increases inflammation. The study by Podkowińska et al. [49] focused attention on increased oxidative stress and inflammation in ESRD patients. The combination of elevated Pb levels and oxidative stress can create a synergistic effect, exacerbating cellular damage, including damage to DNA. Pb accumulation in tissues was reported to cause oxidative DNA damage, including strand breakages in workers exposed to Pb [50]. So, the elevated Pb levels in our study may have also caused increased inflammation and DNA damage.

Urinary excretion represents the major route of As elimination. Since a large fraction of absorbed As is filtered in the kidney, it is an important site of As uptake and accumulation. Contamination of drinking water with As has been linked to the development of hypertension and renal injury. Exposure to As was shown to cause albuminuria and proteinuria, but it did not enhance other outcomes (e.g., anemia, hyperkalemia, hypocalcemia) that are associated with CKD. Although exposure to As appears to cause renal injury, there is not a clear association between As exposure and the development of CKD [51,52]. In a previous study, total As level was associated significantly with CKD in a dose–response manner, especially in participants with a total As level greater than 20.74 ppb, compared with 11.78 microg/g creatinine or less [53]. Two other studies have also focused on high blood-As levels in ESRD patients. The study by Palaneeswari et al. [54] compared the blood As levels in 50 ESRD patients with 50 healthy controls, and they found significantly higher levels of As in ESRD patients, similar to our results. However, they did not compare the ESRD patients with regard to hemodialysis status. Our results have shown that As levels were significantly higher than in healthy controls, although the As levels between the two patient groups were similar. Therefore, we can suggest that ESRD patients have high levels of As, which might be one of the causes of renal failure.

Oxidative stress is known to take place in the pathophysiology of CKD progression and various types of kidney diseases. The exact pathophysiological mechanisms underlying chronic tubulointerstitial nephropathy have not yet been fully elucidated. Oxidative stress, fibrosis, and inflammation are thought to play a role in the development and progression of ESRD. The oxidation of biomolecules by ROS starts very early in CKD, progresses in parallel with the deterioration in kidney function, and is further exacerbated in ESRD [55,56]. Numerous studies indicated that oxidative stress significantly increased in patients with advanced renal impairment, but this state was exacerbated by hemodialysis [57,58,59]. In our study, the levels of MDA and 8-OHdG increased in ESRD patients compared with healthy controls, which indicates increases in oxidative stress, with an expected outcome. Similar to the previous studies, the main antioxidant enzymes, SOD, CAT, and GPx, significantly increased in ESRD patients compared with healthy controls. Though the differences were not statistically significant between ESRD patients undergoing dialysis and ESRD patients not on dialysis, the levels of CAT and GPx were highest in the hemodialysis group in our study. These increases in antioxidant enzyme levels in ESRD patients may indicate that the body’s antioxidant protection mechanism against increased oxidative stress also increases.

Oxidative stress is often increased in patients with ESRD, due to various factors, including inflammation, uremic toxins, and the dialysis process itself. The end product of lipid peroxidation, MDA, was also elevated in ESRD patients compared to healthy controls. In our study groups, MDA levels in ESRD patients undergoing dialysis were significantly higher than in the group not undergoing dialysis. This points out to an exacerbated oxidative response in this patient group. Significant differences found between three study groups can be accepted as an indicator of increased oxidative stress and nucleophilic ROS attacks in ESRD patients, especially in the group undergoing dialysis.

A previous study observed that ESRD patients on dialysis exhibited significantly increased oxidative stress compared with pre-dialysis CKD stage 4. This increase can be attributed to various factors. Typically, physicians impose strict dietary restrictions on dialysis patients, avoiding the consumption of fruits and vegetables that are rich in potassium, to prevent hyperkalemia, thus resulting in a reduced intake of dietary antioxidants, vitamins, and flavonoids. Also, a high number of antioxidants (such as vitamin C and trace elements) are lost during every hemodialysis session, and the activation of white blood cells, inflammation and ROS overproduction is seen after every dialysis session [60]. The elevated levels of plasma MDA was also reported by Valentini et al. [61].

Oxidative-stress-induced DNA damage can result in DNA single- or double-strand breakage, base modifications, deoxyribose modifications, and DNA cross-linking. Guanine exhibits the lowest redox potential among the DNA bases, and is therefore the main target for oxidative damage in the DNA. 8-Oxo-7,8-dihydro-2-deoxyguanosine (8-oxodG) is the most frequent modification of guanine, and is often measured when oxidative DNA damage is to be quantified as a biomarker. In conformity with the data from the cytokinesis-block micronucleus assay and the comet assay, analysis of 8-oxodG levels shows an increase in dialysis patients. Similar to these data, our findings support the previous studies which confirmed elevated levels of 8-oxodG in chronic-kidney-disease patients [62,63]. In our study, both 8-oxodG levels and DNA damage increased in ESRD patients undergoing dialysis. These levels were lower in ESRD patients than in those not undergoing dialysis, but still higher than in the healthy control group. The significant differences found between the three study groups can be accepted as an indicator of increased oxidative stress and nucleophilic ROS attacks in ESRD patients, especially in the group undergoing dialysis.

It has been reported in various studies that genotoxic damage increases in chronic renal failure, in both the predialysis and the dialysis phase [64,65,66,67,68]. In a study with 24 age- and sex-matched healthy subjects, 22 non-dialyzed patients with advanced renal failure, and 42 chronic peritoneal-dialysis patients, a profound increase in 8-OHdG levels in peripheral leukocyte DNA occurred during chronic renal failure, gradually increasing with disease progression [66]. The severity of renal disease is linked to DNA damage in the patients undergoing dialysis [67]. Kan et al. [65] have also found an increase in DNA breakage in the lymphocytes of 36 hemodialysis patients compared with healthy controls, using the alkaline comet assay, similar to our findings.

In the study by Gandhi et al. [68] with 55 ESRD patients undergoing dialysis and 39 healthy controls, increases in DNA damage and micronucleus frequency were shown in patients using the single-cell gel electrophoresis assay and the micronucleus test, respectively. In this study, it was reported that time-on-medication and time-on-dialysis were correlated with genetic damage. The significant increases in DNA damage and micronucleus frequency seen in ESRD patients was attributed to the accumulation of uremic toxic substances. It was suggested that DNA and chromosome damage might be useful prognostic biomarkers to initiate timely intervention against comorbidities in ESRD patients [68].

The renal disease state, oxidative stress, inflammatory responses, and treatment procedure may have detrimental effects on genetic material. Tung and Gandi [69] investigated DNA damage using the comet assay in peripheral blood leukocytes of patients (n = 200) with stage V Chronic Kidney Disease (those receiving dialysis, and those recommended but not yet started dialysis, and compared the results with healthy controls (n = 210). It was reported that patients receiving the twice-weekly dialysis regimen had significantly higher percent tail DNA and Damage Index compared to those without dialysis and the once-weekly dialysis group. This study indicated that dialysis-induced mechanical stress and blood-dialysis device membrane interactions were possible contributors to the increases in DNA damage. These findings pointed to the need to improve and develop interventional therapies to delay disease progression and associated comorbidities, in order to improve the life expectancy of patients with kidney disease [69].

The specific levels of antioxidant enzymes in ESRD patients can vary depending on the individual, the stage of ESRD, and the presence of other complications. In some cases, their levels may be elevated as a response to increased oxidative stress, while in other cases they may be decreased, due to various factors, including reduced antioxidant capacity. In this study, levels of both metals were higher in patients with disease duration over 10 years. It is known that heavy metals are excreted renally, and functional loss of nephrons may cause accumulation of these metals in the body. This might be a sign of the accumulation of heavy metals in ESRD patients over a long period of time, pointing to a diminished elimination of toxic metals from kidneys. Oxidative-stress parameters did not show any significant difference when compared according to disease duration. There were, however, differences in MDA levels between patients with short and long disease duration, and also with medium and long disease duration. MDA levels were highest in patients having the disease for over 10 years. It is known that lipids are one of the main targets of ROS and heavy metals. So, increased lipid peroxidation over time points to increased oxidative stress over a time period. In the study by Drai et al. [70], MDA levels were also elevated in long-term hemodialysis patients. The same situation was also present with respect to 8-OHdG levels. These findings can suggest an increased lipid peroxidation and DNA damage over an elongated time course of the disease. It is clear that genotoxic effects increase as the duration of the disease increases.

Our study does also suggest some limitations. One of them can be listed as limited sample size. The participants were from different occupational backgrounds, so the toxic effects of heavy metals were not standardized according to different occupations of patients. Also, the diet and smoking status of the participants were not standardized. More detailed studies with large numbers of patients are necessary.

## 5. Conclusions

There are very limited detailed studies focused on oxidative-stress-related genotoxic events in ESRD patients undergoing dialysis and on those not on dialysis. In our study, genotoxicity, oxidative stress, and heavy-metal levels (except mercury) increased significantly in ESRD patients. The Al and Cd levels, DNA damage, 8-OHdG and MDA levels significantly increased and GSH levels significantly decreased in the patients undergoing dialysis compared with those not having dialysis.

In conclusion, our study is a pioneer report discussing the role of heavy metals, oxidative stress and genotoxic damage in patients with end-stage renal disease. It is assumed that these changes may play an important role in the progression of renal damage. The approaches to reducing oxidative-stress-related DNA damage and heavy-metal load in ESRD patients are recommended.

## Figures and Tables

**Table 1:** Characteristics of the study groups.

	ESRD Patients on Dialysis	ESRD Patients without Dialysis	Healthy Controls
	(*n* = 43)	(*n* = 31)	(*n* = 41)
Age (years)	59.43 ± 16.88	67.55 ± 17.69	61.73 ± 18.06
	(32–83)	(37–87)	(33–87)
Gender [*n* (%)]			
Male	27 (62.79%)	14(45.16%)	21(51.22%)
Female	16 (37.21%)	17 (54.83%)	19 (48.78%)
Body mass index (BMI) (kg/m^2^)	2.46 ± 0.48	2.79 ± 0.52	2.74 ± 0.53
	(0.53–3.29)	(1.85–4.30)	(1.76–4.10)
Smoking status [*n* (%)]			
Non-smoker	38 (88.37%)	26 (83.87%)	37 (87.57%)
Smoker	5 (11.53%)	5 (16.13%)	4 (12.43%)
Cigarettes/day *	2.09 ± 6.00	3.33 ± 7.58	2.50 ± 8.09
	(10–20)	(20–20)	(20–40)
Alcohol intake [*n* (%)]			
No	43 (100%)	31 (100%)	41 (100%)
Yes	0 (0%)	0 (0%)	0 (0%)
Diabetes Mellitus [*n* (%)]			
No	20 (46.6%)	16 (51.6%)	-
Yes	23 (53.4%)	15 (48.4%)	
Hypertension [*n* (%)]			
No	11 (25.5%)	11 (35.5%)	-
Yes	32(74.5%)	20 (66.5%)	
Cardiac failure [*n* (%)]			
No	3 (6.97%)	3 (9.67%)	-
Yes	40 (93.03%)	28 (90.33%)	
Duration of renal disease (years)	8.16 ± 3.69	5.07 ± 1.70	-
	(2–16)	(2–18)	

* In both groups, individuals who smoked for at least 1 year were considered smokers. n: number of subjects. The values are given as mean ± standard deviation (min–max).

**Table 2:** Hemogram of the study groups.

Parameters	ESRD Patients on Dialysis (n = 43)	ESRD Patients without Dialysis (n = 31)	Healthy Controls (n = 41)	*p* ^a^	*p* ^b^	*p* ^c^
WBC (10^3^/µL)	9.56 ± 4.92 (3.40–28.50)	8.75 ± 4.33 (3.80–26.20)	8.65 ± 7.38 (1.30–49.50)	0.486	0.763	0.726
RBC (10^6^/µL)	3.52 ± 0.63 (2.23–4.78)	3.95 ± 0.57 (2.75–5.31)	4.41 ± 0.80 (2.45–6.10)	<0.001 *	0.004 *	0.012 *
Hb (g/dL)	10.44 ± 1.84 (6.20–14.60)	11.44 ± 1.56 (8.20–16.20)	12.83 ± 2.86 (3.30–18.0)	<0.001 *	0.007 *	0.048 *
PLT (10^3^/µL)	208.05 ± 76.38 (95–368)	227.40 ± 65.80 (39–345)	234.66 ± 70.52 (108–366)	0.109	0.455	0.447
HCT (%)	31.68 ± 5.58 (18.10–43.20)	34.48 ± 4.97 (24.70–49.60)	39.00 ± 6.29 (22.90–51.80)	<0.001 *	<0.001 *	0.044 *
MCV (fL)	89.64 ± 14.65 (74.9–100.9)	87.62 ± 4.99 (76.80–100.60)	88.81 ± 4.97 (78.20–97.60)	0.246	0.966	0.255
MCH (pg)	29.56 ± 1.84 (23.40–33.80)	29.00 ± 2.18 (24.40–32.60)	29.07 ± 4.69 (3.80–33.70)	0.491	0.945	0.562
MCHC (g/dL)	33.00 ± 1.06 (31.10–36.50)	32.51 ± 2.69 (23.20–35.10)	33.52 ± 1.03 (31.00–35.70)	0.166	0.013 *	0.207
RDW-SD (fL)	49.08 ± 15.33 (39.40–419)	46.98 ± 6.60 (39.40–66.90)	46.41 ± 11.93 (0.00–84.0)	0.112	0.786	0.216
RDW-CV (%)	10.90 ± 6.96 (12.08–18.80)	15.23 ± 2.36 (12.70–22.10)	14.82 ± 2.85 (11.70–25.40)	0.683	0.438	0.685
MPV (fL)	8.95 ± 1.13 (6.80–11.30)	9.05 ± 0.92 (7.00–11.00)	9.07 ± 1.27 (6.10–11.40)	0.638	0.970	0.629
NEU (%)	63.81 ± 24.46 (3.22–96.60)	70.02 ± 11.47 (52.50–95.80)	65.62 ± 17.78 (5.45–91.50)	0.638	0.970	0.629
MO (%)	6.65 ± 3.77 (0.32–14.00)	7.68 ± 2.29 (2.90–12.20)	6.76 ± 2.34 (0.50–13.00)	0.858	0.226	0.160
LYM (%)	15.06 ± 10.95 (0.80–43.50)	19.35 ± 9.51 (0.80–36.20)	23.12 ± 13.28 (2.23–53.20)	0.002 *	0.185	0.108
EOS (%)	2.18 ± 2.08 (0.01–7.60)	2.45 ± 2.01 (0.10–7.60)	1.53 ± 1.42 (0.00–6.60)	0.123	0.055	0.610
BASO (%)	0.59 ± 0.40 (0.02–1.80)	0.64 ± 0.33 (0.10–1.50)	0.56 ± 0.34 (0.03–1.50)	0.708	0.274	0.443

The values are given as mean ± standard deviation (min–max). BASO, basophils; EOS, eosinophils; ESRD, end-stage renal disease, HCT, hematocrit; Hb, hemoglobulin; LYM, lymphocytes; MCH, mean corpuscular hemoglobin; MCHC, mean corpuscular hemoglobin concentration; MCV, mean corpuscular volume; MO, monocytes; MPV, mean platelet volume; NEU, neutrophils; PLT, platelet; RBC, red blood cells; RDW-SD, red-cell distribution width-standard deviation; WBC, white blood cells. ^a^ *p* < 0.05, ESRD patients on dialysis compared with healthy controls; ^b^ *p* < 0.05, ESRD patients without dialysis compared with healthy controls, ^c^ *p* < 0.05, ESRD patients on dialysis compared with ESRD patients without dialysis; *, statistically significant.

**Table 3:** Biochemical parameters of the study groups.

Parameters	ESRD Patients on Dialysis (n = 43)	ESRD Patients without Dialysis (n = 31)	Healthy Controls (n = 41)	*p* ^a^	*p* ^b^	*p* ^c^
AST (U/L)	22.85 ± 19.40 (7–107)	25.52 ± 19.99 (6–287)	25.38 ± 14.42 (4–74)	0.719	0.245	0.127
ALT (U/L)	15.71 ± 10.98 (4–64)	19.97 ± 10.81 (5–57)	28.76 ± 39.61 (5–192)	0.022 *	0.221	0.363
Glucose (mg/dL)	127.59 ± 74.40 (52–392)	136.11 ± 71.76 (53–321)	129.00 ± 85.88 (71–477)	0.768	0.941	0.867
Natrium (Na) (mmol/L)	136.14 ± 5.08 (117–149)	137.70 ± 5.24 (126–143)	136.89 ± 37.58 (129–146)	0.332	0.175	0.633
Potassium (K) (mmol/L)	4.90 ± 1.03 (3.83–6.23)	4.62 ± 0.69 (3.08–6.09)	4.25 ± 0.89 (3.46–5.80)	0.159	0.060	0.558

The values are given as mean ± standard deviation (min–max). ALT, alanine aminotransferase; AST, aspartate aminotransferase. ^a^ *p* < 0.05, patients on dialysis compared with healthy controls; ^b^ *p* < 0.05, patients without dialysis compared with healthy controls, ^c^ *p* < 0.05, patients on dialysis compared with patients without dialysis; *, statistically significant.

**Table 4:** Renal functions of the study groups.

Parameters	ESRD Patients on Dialysis (n = 43)	ESRD Patients without Dialysis (n = 31)	Healthy Controls (n = 41)	*p* ^a^	*p* ^b^	*p* ^c^
Creatinine (mg/dL)	6.36 ± 3.60 (0.58–15.30)	2.70 ± 1.60 (0.76–7.66)	1.44 ± 1.46 (0.53–8.81)	<0.001 *	0.049 *	<0.001 *
Urea (mg/dL)	120.62 ± 48.70 (32.0–225.0)	98.00 ± 51.18 (31.0–215.0)	50.82 ± 36.76 (21.0–205.0)	<0.001 *	<0.001 *	0.023 *
GFR (mL/min/1.73 m^2^) **	12.29 ± 10.90 (4.0–52.0)	28.67 ± 13.86 (6.0–80.0)	69.50 ± 31.07 (5.58–136.0)	<0.001 *	<0.001 *	0.001 *

The values are given as mean ± standard deviation (min–max). ^a^ *p* < 0.05, ESRD patients on dialysis compared with healthy controls; ^b^ *p* < 0.05, ESRD patients without dialysis compared with healthy controls, ^c^ *p* < 0.05, ESRD patients on dialysis compared with ESRD patients without dialysis; *, statistically significant. **, Estimated glomerular filtration rate (GFR), an indicator of renal function, was calculated using the CKD-EPI equation [26,27].

**Table 5:** Heavy-metal levels of the study groups.

Parameters	ESRD Patients on Dialysis	ESRD Patients without Dialysis	Healthy Controls	*p* ^a^	*p* ^b^	*p* ^c^
Al (ppb)	n = 11/23 (47.8%) 3.58 ± 0.72 (2.94–5.50)	n = 1/28 (3.57%) 3.80 (3.80–3.80)	n = 0/32 (0.0%) 0 ND	-	-	0.780
Cd (ppb)	n = 17/23 (73.9%) 8.07 ± 15.76 (0.05–49.36)	n = 21/28 (75.0%) 0.67 ± 0.98 (0.05–4.25)	n = 17/32 (53.1%) 0.16 ± 0.12 (0.05–0.45)	0.007 *	0.851	0.049 *
Pb (ppb)	n = 10/23 (43.5%) 13.57 ± 9.03 (3.34–35.29)	n = 11/28 (39.3%) 20.41 ± 15.11 (5.67–52.25)	n = 1/32 (3.12%) 0.38 (0.38–0.38)	<0.001 *	<0.001 *	0.225
As (ppb)	n = 23/23 (100%) 4.35 ± 0.93 (2.85–6.35)	n = 26/28 (92.9%) 3.86 ± 1.67 (2.19–10.45)	n = 32/32 (100%) 2.35 ± 0.78 (1.23–5.46)	<0.001 *	<0.001 *	0.132
Hg (ppb)	ND	ND	ND	-	-	-

The values are given as mean ± standard deviation (min–max). ^a^ *p* < 0.05, ESRD patients on dialysis compared with healthy controls; ^b^ *p* < 0.05, ESRD patients without dialysis compared with healthy controls, ^c^ *p* < 0.05, ESRD patients on dialysis compared with ESRD patients without dialysis; *, statistically significant. ND: non-detectable, n = number of samples. 1 ppm, 1 µg/L. Al, aluminum; Cd, cadmium; Pb, lead; As, arsenic; Hg, mercury.

**Table 6:** Oxidative-stress parameters of the study groups.

Parameters	ESRD Patients on Dialysis (n = 43)	ESRD Patients without Dialysis (n = 31)	Healthy Controls (n = 41)	*p* ^a^	*p* ^b^	*p* ^c^
SOD (ng/mL)	36.12 ± 37.32 (2.57–128.5)	20.59 ± 15.61 (6.89–68.27)	12.44 ± 6.36 (4.20–28.93)	<0.001 *	0.051 *	0.011 *
CAT (ng/mL)	34.54 ± 20.15 (13.83–106.8)	37.76 ± 26.91 (18.04–151.1)	25.40 ± 7.88 (18.04–151.1)	0.046 *	0.021 *	0.530
GPx (ng/mL)	55.80 ± 33.31 (19.93–136.3)	57.87 ± 21.14 (12.47–124.0)	39.62 ± 12.40 (10.70–60.51)	0.010 *	0.007 *	0.746
GSH (nmol/L)	1.54 ± 0.86 (0.14–3.52)	2.07 ± 0.77 (1.01–3.84)	4.83 ± 3.60 (1.60–15.51)	<0.001 *	0.011 *	0.355
MDA (nmol/L)	32.97 ± 25.97 (9.15–90.80)	21.11 ± 10.57 (9.15–58.04)	11.49 ± 6.05 (1.17–21.09)	<0.001 *	0.041 *	0.010 *
8-OHdG (ng/mL)	30.45 ± 27.64 (8.41–98.20)	15.36 ± 12.60 (6.99–71.29)	7.43 ± 3.01 (0.75–14.94)	<0.001 *	0.048 *	0.002 *

The values are given as mean ± standard deviation (min–max). SOD, superoxide dismutase; CAT, catalase; GPx, glutathione peroxidase; GSH, glutathione; MDA, malondialdehyde; 8-OHdG: 8-hydroxy-2′-deoxyguanosine. ^a^ *p* < 0.05, ESRD patients on dialysis compared with healthy controls; ^b^ *p* < 0.05, ESRD patients without dialysis compared with healthy controls, ^c^ *p* < 0.05, ESRD patients on dialysis compared with ESRD patients without dialysis; *, statistically significant. 1 ppm, 1 µg/L.

**Table 7:** Changes in heavy metals, oxidative stress, and DNA-damage parameters, depending on the duration of renal disease.

Parameters	Short Duration 2–5 Years (n = 29)	Medium Duration 6–9 Years (n = 28)	Long Duration ≥10 Years (n = 17)
Heavy-metal levels			
Al (ppb)	3.13 ± 1.81 (2.94–3.31) ^a^	3.60 ± 0.30 (3.17–3.81)	3.76 ± 0.93 (2.94–5.50)
Cd (ppb)	0.75 ± 1.03 (0.05–4.25) ^a^	3.14 ± 9.29 (0.05–35.31)	13.42 ± 20.66 (0.12–49.36)
Pb (ppb)	18.22 ± 15.38 (5.67–52.25)	20.22 ± 14.68 (4.22–44.74)	12.15 ± 5.42 (3.34–18.32)
As (ppb)	3.84 ± 1.03 (2.41–6.27)	4.12 ± 1.83 (2.19–10.45)	4.76 ± 0.84 (3.82–6.35)
Oxidative-stress markers			
SOD (ng/mL)	24.32 ± 22.81 (6.89–158.6)	25.81 ± 27.20 (6.34–127.3)	37.27 ± 36.89 (2.57–112.1)
CAT (ng/mL)	34.13 ± 15.00 (21.28–89.17)	36.87 ± 28.55 (18.04–151.1)	39.30 ± 28.14 (13.83–106.8)
GPx(ng/mL)	57.34 ± 28.88 (12.47–124)	50.86 ± 22.93 (27.09–115.1)	66.30 ± 37.81 (27.59–136.3)
GSH (nmol/L)	1.72 ± 0.83 (0.55–3.84)	1.85 ± 0.84 (0.14–3.52)	1.63 ± 1.01 (0.23–3.46)
MDA (nmol/L)	23.49 ± 15.13 (12.57–83.83) ^a^	23.43 ± 18.65 (9.15–90.8) ^b^	36.74 ± 26.49 (12.75–87.52)
8-OHdG (ng/mL)	17.92 ± 16.54 (6.98–98.2) ^a^	20.11 ± 19.95 (9.001–88.3) ^b^	32.94 ± 25.66 (8.50–81.63)
DNA damage			
Tail intensity	5.61 ± 3.35 (1.45–13.29) ^a^	5.44 ± 3.43 (1.19–16.61) ^b^	8.28 ± 3.45 (2.68–12.45)

The values are given as mean ± standard deviation (min–max). DNA damage is expressed as tail intensity (% DNA in the tail) in the lymphocytes. n: number of subjects. Al, aluminum; Cd, cadmium; Pb, lead; As, arsenic. SOD, superoxide dismutase; CAT, catalase; GPx, glutathione peroxidase; GSH, glutathione; MDA, malondialdehyde; 8-OHdG, 8-hydroxy-2′-deoxyguanosine. ^a^ *p* < 0.05, short duration compared with long duration; ^b^ *p* < 0.05, medium duration compared with long duration.

## Data Availability

The data presented in this study are available on request from the corresponding author. The data are not publicly available due to ethical restrictions.
